# Help-seeking behaviors for female sexual dysfunction: a cross sectional study from Iran

**DOI:** 10.1186/1472-6874-9-3

**Published:** 2009-02-27

**Authors:** Mariam Vahdaninia, Ali Montazeri, Azita Goshtasebi

**Affiliations:** 1 Iranian Institute for Health Sciences Research (IHSR), ACECR, Tehran, Iran; 2 Tarbiat Modarres University, Tehran/Iran

## Abstract

**Background:**

Female sexual dysfunctions (FSD) are prevalent multifactor problems that in general remain misdiagnosed in primary health care. This population-based study investigated help-seeking behaviors among women with FSD in Iran.

**Methods:**

This was a cross sectional study carried out in Kohgilouyeh-Boyer-Ahmad province in Iran. Using quota sampling all sexually active women aged 15 and over registered in primary health care delivery centers were studied. Experience of sexual problems was assessed using an ad-hoc questionnaire (Female sexual dysfunction: help-seeking behaviors survey) containing 14 items. Trained female nurses interviewed all participants after a verbal informed consent. Data were analyzed in a descriptive manner.

**Results:**

In all 1540 women were studied. Of these, 786 (51%) cases had experienced at least one of the FSD problems. Results showed that 35.8% of women with FSD had sought no professional help and the most reasons for not seeking help were identified as: 'time constraints' and believing that it 'did not occur to me' (39.1 and 28.5% respectively). Sixty one percent of women who sought help for FSD reported that 'doctor gave me a definite diagnosis' and 'a definite treatment plan was given' in 57% of cases.

**Conclusion:**

The study findings indicated that FSD problems were prevalent and many women did not seek help for their problem. Finding 'time constraints' and believing that the problem 'did not occur to me' as the most cited reasons for not seeking help might facilitate to understand potential barriers that exist in recognition and treatment of the female sexual dysfunctions. Since FSD might have a negative impact on interpersonal relationships and women's quality of life, it seems that there is need to address the problem both at local and national primary health care services.

## Background

Female sexual dysfunctions (FSD) are gender-related prevalent multifactor problems. Studies on epidemiologic aspects of these disorders are sparse. Also, inconsistent definitions of FSD, characteristics of different populations and sample size restrictions make difficult the comparability of FSD estimates in an international level [1]. A study on sexual problems among men and women aged 40–80 years from 29 countries found that about half of all sexually active women had experienced at least one sexual problem [2]. However the study sample was not representative of each country's adult population.

Studies have shown that FSD might cause a negative impact on interpersonal relationships and quality of life. It is argued that intimacy in sexual relationships and favor sexual function has an essential role in maintaining psychological satisfaction and good quality of life [3-6]. Similarly studies indicated that satisfaction with emotional and physical sexual relationships is predictive of subjective sexual well being in elderly [7]. In addition, sexual problems are considered as a vicious circle in infertility and reproductive disorders. Childlessness may be the result of a neglected sexual dysfunction that could be elucidated in medical consultation. Also stress due to infertility may interact with couples' or individuals' sexuality and result in arousal difficulties [8].

Evidence suggests that despite the high prevalence of FSD disorders these problems frequently go undetected in primary health care services [9,10]. Improvement of knowledge and skills in general practice as well as contextual changes are suggested solutions for better management of sexual dysfunctions [10]. Many people like to discuss their sexual problems with doctors during their routine visits [11], but the question remains whether the primary healthcare professionals are prepared to deal with such problems.

As part of a study on women's health, an investigation was carried out to assess FSD prevalence and help-seeking behaviors among sexually active women in Kohgilouyeh-Boyer-Ahmad province, in Iran. The province is located in southwest of Iran and has 700,000 inhabitants. In general, the province is known as a deprived area but its primary healthcare system is very well organized and most people use the services for their health-related problems.

## Methods

### Design and data collection

This was a cross-sectional study carried out in year 2005. A team of trained female nurses interviewed all participants after a verbal informed consent.

### Questionnaire

The questionnaire was derived from the literature [2,12]. Two psychiatrists and a clinical psychologist assessed and confirmed its content validity. Then the questionnaire was pilot tested to examine if the questions were clear and acceptable. Considering cultural issues and that the survey was being conducted in a deprived area, some items were amended or reduced. The final questionnaire consisted of 14 items in three parts: demographic characteristics (3 items), experience of FSD (6 items), and 5 items on help-seeking behaviors [Additional file 1]. Sexual problems were defined as: desire disorder (DD), arousal disorder (AD), lubrication disorder (LD), orgasmic disorder (OD), satisfaction disorder (SD) and pain disorder (PD). Experience of any of the defined sexual problems was assessed by a single self-reported question during the last 3 months. The level or degree of the disorders was rated on a 4-point scale. For analysis response categories were combined to yield either "existence of the problems" or "lack of the disorder".

Help-seeking behavior among women with FSD was assessed by asking women: "Have you ever sought any help from healthcare services for your problem?" A list of services was provided and more than one service could be indicated. Subjects who had not sought professional consultation were asked about the reasons. Also they were asked, "If they are willing to have treatment now?" Finally, attitudes and beliefs towards the medical consultation in women who sought help for their problems also were assessed.

### The sample

From a pilot study we estimated that for the main study there is need for a sample of 1500 sexually active women. The study population consisted of all sexually active married women aged 15 and over. Exclusion criteria were pregnancy, having chronic diseases and mental disorders. To select a representative sample of the population, a stratified multi stage area sampling was applied. Every woman registered in primary healthcare centers had the same probability to be sampled.

### Analysis

Data were analyzed using SPSS 13 in a descriptive fashion and restricted to those who had FSD.

### Ethics

The study was approved by the Iranian Academic Center for Education, Culture and Research (ACECR). All women gave their oral informed consent.

## Results

In all 1540 women were studied. Of these, 786 (51.%) women were found to experience FSD. The mean age of women with FSD was 33.2 years (SD = 9.4%), and mostly were housewife (86.4%). There were significant differences between educational and employment status of women with and without FSD indicating that those with FSD were less educated and more housewife. Table 1 shows the demographic characteristics of the study sample.

**Table 1:** Demographic characteristics of the study sample

	**Total sample**	**With FSD**	**Without FSD**	P*
	**Number (%)**	**Number (%)**	**Number (%)**	

**Age (groups)**	(n = 1540)	(n = 786)	(n = 754)	0.4

≤20	90 (5.8)	54 (6.9)	36 (4.8)	

21–30	591 (38.5)	292 (37.2)	299 (39.7)	

31–40	552 (35.8)	278 (35.3)	274 (36.3)	

41–50	239 (15.5)	128 (16.3)	111 (14.7)	

≥51	68 (4.4)	34 (4.3)	34 (4.5)	

Mean (SD)	33.2 (9.4)	33.2 (9.4)	33.2 (9.3)	

**Educational Status**	(n = 1470)	(n = 752)	(n = 718)	< 0.001

Illiterate	236 (16.1)	126 (16.8)	110 (15.3)	

Primary	428 (29.1)	242 (32.2)	186 (25.9)	

Secondary	271 (18.4)	132 (17.6)	139 (19.4)	

High school	378 (25.7)	196 (26.1)	182 (25.3)	

University	157 (10.7)	56 (7.3)	101 (14.1)	

Mean (SD)	7.1 (4.8)	6.7 (4.6)	7.6 (4.9)	

**Employment Status**	(n = 1540)	(n = 786)	(n = 754)	0.03

Housewife	1301 (84.5)	679 (86.4)	622 (82.5)	

Others	239 (15.5)	107 (13.6)	132 (17.5)	

*χ^2 ^test

Age distribution of women with FSD and its items is shown in Table 2. Orgasmic disorder (OD) was found to be the most prevalent disorder among women (38.0%) and lubrication disorder (LD) was the least reported one (21.0%). In youngest age group (≤20 years) the most observed FSD disorder was pain disorder (PD, 37%) and arousal disorder (DD) was the most prevalent disorder (61.8%) among older age group (≥51 years).

**Table 2:** Age distribution of FSD

	**≤20 (n = 54)**	**21–30 (n = 292)**	**31–40 (n = 278)**	**41–50 (n = 128)**	**≥51 (n = 34)**	**All (n = 786)**
	**Number (%)**	**Number (%)**	**Number (%)**	**Number (%)**	**Number (%)**	**Number (%)**

**DD**						

No	40 (74.1)	210 (71.9)	171 (61.5)	84 (65.6)	13 (38.2)	518 (65.9)

Yes	14 (25.9)	82 (28.1)	107 (38.5)	44 (34.4)	21 (61.8)	268 (34.1)

**AD**						

No	43 (79.6)	200 (68.5)	179 (64.4)	82 (64.1)	21 (61.8)	525 (66.8)

Yes	11 (20.4)	92 (31.5)	99 (35.6)	46 (35.9)	13 (38.2)	261 (33.2)

**LD**						

No	47 (87.0)	234 (80.1)	211 (75.9)	103 (80.5)	26 (76.5)	621 (79.0)

Yes	7 (13.0)	58 (19.9)	67 (24.1)	25 (19.5)	8 (23.5)	165 (21.0)

**OD**^3^						

No	36 (66.7)	179 (61.3)	171 (61.5)	80 (62.5)	21 (61.8)	487 (62.0)

Yes	18 (33.3)	113 (38.7)	107 (38.5)	48 (37.5)	13 (38.2)	299 (38.0)

**PD**						

No	34 (63.0)	189 (64.7)	193 (69.4)	92 (71.9)	28 (82.4)	536 (68.2)

Yes	20 (37.0)	103 (35.3)	85 (30.6)	36 (28.1)	6 (17.6)	250 (31.8)

**SD**						

No	39 (72.2)	182 (62.3)	179 (64.4)	79 (61.7)	21 (61.8)	500 (63.6)

Yes	15 (27.8)	110 (37.7)	99 (35.6)	49 (38.3)	13 (38.2)	286 (36.4)

DD: Desire Disorders, AD: Arousal Disorders, LD: Lubrication Disorders, OD: Orgasmic Disorders, PD: Pain Disorders, SD: Satisfaction Disorders

Table 3 presents the help-seeking behavior and reasons for not seeking help for FSD. In general 505 women (64.2%) had sought professional consultation and 281 cases (35.8%) had sought none. Most of the women had sought help from a gynecologist or a general practitioner (33.2 and 13.9% respectively). The most reported reasons for not seeking help were: 'I had time constraints' (39.1%) and 'It did not occur to me' (28.5%). Also, 'Being ashamed to speak about the problem' and 'doctor can't help me' were cited by 9.6% of women respectively. However, 56.9% of these cases had willing to have treatment for their disorder.

**Table 3:** Help-seeking patterns for FSD among women

	**Number**	**%**
**Help-seeking for FSD (n = 786)**		

None	281	35.8

From gynecologist	261	33.2

From general practitioner	109	13.9

From psychiatrist	11	1.4

From more than one health professionals	124	15.7

**Attitudes and beliefs for not seeking help for FSD (n = 281)**		

I am ashamed to speak about it	27	9.6

Doctor can not help me	27	9.6

I had time constraints	110	39.1

It did not occur to me	80	28.5

I was not asked about my problem during my routine visit(s)	5	1.8

Responding to more than one choices	32	11.4

**Are you willing to have treatment now? (n = 281)**		

No	62	22.1

Yes	160	56.9

I am not sure	59	21.0

Attitudes and beliefs towards medical consultations in women who had sought help for FSD is shown in Table 4. Doctor had given genital examination in most of the cases (69.0%). Based on the results 60.8% of women believed that their doctor gave them a definite diagnosis. Also 57.0% of subjects believed that "doctor gave them a definite treatment plan".

**Table 4:** Attitudes and beliefs towards the medical consultation in women who sought help for FSD

	**Agree**	**Disagree**
	**Number (%)**	**Number (%)**

Doctor listened carefully to me (n = 485)	396 (81.6)	89 (18.4)

Doctor gave me a genital examination (n = 471)	325 (69.0)	146 (31.0)

Doctor ordered lab tests (n = 485)	342 (70.5)	143 (29.5)

Doctor asked about my emotional and mental status (n = 470)	231 (49.0)	239 (51.0)

Doctor enquired about the quality of my sex life (n = 463)	241 (52.0)	222 (48.0)

Doctor gave me a definite diagnosis (n = 472)	287 (61.0)	185 (39.0)

Doctor gave me a definite treatment plan (n = 477)	272 (57.0)	205 (43.0)

The relationships between demographic characteristics and help-seeking behavior for FSD women were examined. No significant associations were observed between women who sought and who did not seek help for their FSD problems regarding their age, education and employment.

## Discussion

This study provided data on FSD prevalence and its help-seeking behavior in sexually active women aged 15 years old and over in Kohgilouyeh-Boyer-Ahmad province/Iran. In this study sexual problems that were experienced in the last 3 months were considered as "dysfunction". This allowed minimizing the false positive responses and obtaining more valid data on FSD. Also most of the women were interested in participating in the study and the overall response rate was 92%.

Fifty one percent of the whole study population (786/1540) had experienced at least one of the sexual problems and the most observed dysfunction was "orgasmic disorder" (Table 2). High prevalence of sexual problems among women is reported in different populations [5,11-16].

The results showed that 64.2% of women had attempted to seek medical consultation for their disorder. The Global Study on Sexual Attitudes and Behaviors (GSSAB) in 29 countries showed that although about half of all sexually active respondents had experienced at least one sexual problem, less than 18.8% of women had sought medical help for their sexual problems [2]. It is argued that seeking help for sexual problems reflects awareness of the availability of advice and treatment for these disorders [17].

In this study most women had referred to a gynecologist (33.2%) and a general practitioner (13.9%) While a small proportion of women with FSD (1.4%) had sought help from a psychiatrist. In addition 15.4% of women had sought help from more than one health professionals (Table 3). The latter implies that women had been dissatisfied with their initial management and had sought further consultations from other health professionals. Moreover the observed diverse pattern of help-seeking for FSD is suggestive that there is not a definite diagnosis and treatment plan for FSD in primary health care services in Iran. The GSSAB has found that among women an attempt to seek medical help for sexual problems has been 18.8% and about 1–8% of seeking psychological help (psychiatrist, psychologist or marriage counselor) has been reported [2].

'Time constraints' and believing that the problem 'did not occur to me' were the most cited reasons for not consulting a doctor for FSD. Also, the belief that 'doctor cannot help me' was reported by 9.6% of women (Table 3). It seems these reasons indicate that women with FSD may not consider their problem serious. Similarly studies have found that not feeling on the severity of the sexual problems may deter individuals discussing their sexual difficulties [18,19]. In addition the idea that 'the sexual problem was a normal part of getting older/being comfortable the way I am' were commonly cited reasons by women in GSSAB study [2].

Other beliefs in respect of not seeking help were: 'Being ashamed to speak about the problem' and 'not being asked by the doctor during routine visits' (Table 3). Similar findings have indicated that doctors in Europe and other countries rarely ask patients about their sexual health during a routine consultation even though patients would appreciate this [2,18,19]. The reluctance to initiate a discussion about sexual health is a dual interaction between patients and doctors. This may be in part affected by patients' barriers such as embarrassment, lack of knowledge and indirect presentation of the disease [10]. On the other hand due to the lack of appropriate medical training on sexual problems, doctors encounter difficulties in the management and treatment of these disorders [20].

We found that 81.6% of women who sought medical consultation agreed that 'doctor listened carefully to them' while 48.% of cases disagreed with 'being asked by doctor about the quality of their sexual life' (Table 4). Also 61.% and 57.% of these women agreed that 'doctor gave them a definite diagnosis and treatment plan', respectively. It seems that physicians often feel unqualified to treat patients with sexual dysfunctions and there is a need for more professional and patient education as well as relevant secondary care services [10,21].

This study provided useful information and indicated the extent of the FSD problems in a deprived area. However, the study has its own limitations. Different domains of the female sexual dysfunctions were assessed only by one question while one might argue there is need to ask more questions for each disorder to elucidate the problem. In addition, since the study relied on women's subjective impression of the FSD treatment (Table 4), the study was limited in conveying the current management of the FSD in Iran.

## Conclusion

The study findings indicated that FSD problems were prevalent and many women did not seek help for their problem. 'Time constraints' and believing that the problem 'did not occur to me' as the most cited reasons for not seeking help might facilitate to understand potential barriers that exist in recognition and treatment of the female sexual dysfunctions. Since FSD might have a negative impact on interpersonal relationships and women's quality of life, it seems that there is need to address the problem both at local and national primary health care services.

## Abbreviations

FSD: Female Sexual Dysfunction; DD: Desire Disorders; AD: Arousal Disorders; OD: Orgasmic Disorders; PD: Pain Disorders; LD: Lubrication Disorders; SD: Satisfaction Disorders; GSSAB: Global Study on Sexual Attitudes and Behaviors.

## Competing interests

The authors declare that they have no competing interests.

## Authors' contributions

MV analyzed the data and wrote the paper. AM contributed to data analysis and editing the final draft. AG designed and carried out the study. All authors read and approved the final manuscript.

## Pre-publication history

The pre-publication history for this paper can be accessed here:



## Supplementary Material

Additional file 1 Female sexual dysfunction: help-seeking behaviors survey. This is a short self-reported questionnaire assessing female sexual dysfunction and its help-seeking behaviors. It also asks about the reasons of not seeking help. Click here for file
