# Supplementary material for: Help-seeking behaviors for female sexual dysfunction: a cross sectional study from Iran
Source: BMC Womens Health. 2009 Feb 27;9:3. doi: 10.1186/1472-6874-9-3 (PMC2651860; doi:10.1186/1472-6874-9-3)
Supplement: Additional file 1 — Female sexual dysfunction: help-seeking behaviors survey. This is a short self-reported questionnaire assessing female sexual dysfunction and its help-seeking behaviors. It also asks about the reasons of not seeking help. [file 1472-6874-9-3-S1.doc]

Female sexual dysfunction: help-seeking behaviors survey

# Iranian Institute for Health Sciences Research (IHSR)

No: ……………

Date: …...………

# DEMOGRAPHIC DATA

| **1. Date of birth…………………………** |  |  |
| --- | --- | --- |
|  |  |  |
| **2. Level of education** |  |  |
|  | □ | Illiterate |
|  | □ | Primary |
|  | □ | Secondary |
|  | □ | University |
|  |  |  |
| **3. Employment status** |  |  |
|  | □ | Housewife |
|  | □ | Others |

SELF-REPORTED SEXUAL PROBLEMS

| During the past 3 months: | | |
| --- | --- | --- |
|  |  |  |
| **4. How would you rate your degree of sexual desire or interest?** |  |  |
|  | □ | None at all |
|  | □ | A little |
|  | □ | Moderate |
|  | □ | High/very high |
|  |  |  |
| **5. How would you rate your level of sexual arousal during sexual activity?** |  |  |
|  | □ | None at all |
|  | □ | A little |
|  | □ | Moderate |
|  | □ | High/very high |
|  |  |  |
| **6. How difficult was it to become wet during sexual activity?** |  |  |
|  | □ | No difficult |
|  | □ | Slightly difficult |
|  | □ | Difficult |
|  | □ | Very difficult/impossible |
|  |  |  |
| **7. How difficult was it for you to reach orgasm?** |  |  |
|  | □ | No difficult |
|  | □ | Slightly difficult |
|  | □ | Difficult |
|  | □ | Very difficult/impossible |
| **8. How satisfied have you been with your overall sexual life?** |  |  |
|  | □ | Satisfied/very satisfied |
|  | □ | Moderately satisfied |
|  | □ | A little satisfied |
|  | □ | Dissatisfied/very dissatisfied |
|  |  |  |
| **9. How would you rate your degree of pain during sexual activity?** |  |  |
|  | □ | None at all |
|  | □ | A little |
|  | □ | Moderate |
|  | □ | High/very high |

### HELP-SEEKING BEHAVIORS

| If you have experienced any of the above problems, please reply to the following questions: | | |
| --- | --- | --- |
| **10. Have you ever sought any help from healthcare services for your problem?** |  |  |
|  | □ | Yes |
|  | □ | No |
|  |  |  |
| **11. If yes, please specify:** |  |  |
| *** You can choose more than one response** |  |  |
|  | □ | From gynecologist |
|  | □ | From general practitioner |
|  | □ | From psychiatrist |
|  |  |  |
| **12. If not, please specify the reason:** |  |  |
| *** You can choose more than one response** |  |  |
|  | □ | I am ashamed to speak about it |
|  | □ | Doctor can not help me |
|  | □ | Because of time constraints |
|  | □ | It did not occur to me |
|  | □ | I was not asked about the problem during my routine visit |
|  |  |  |
| **13.** **Are you willing to have treatment now?** |  |  |
|  | □ | Yes |
|  | □ | No |
|  | □ | I am not sure |
|  |  |  |
| **14. If you have sought help, how was it?** |  |  |
|  | □ | Doctor listened carefully to me |
|  | □ | Doctor gave me a genital examination |
|  | □ | Doctor ordered lab tests |
|  | □ | Doctor asked about my emotional and medical status |
|  | □ | Doctor enquired about the quality of my sexual life |
|  | □ | Doctor gave me a definite diagnosis |
|  | □ | Doctor gave me a definite treatment plan |

Thank you for your cooperation
